# Special orthopaedic geriatrics (SOG) - a new multiprofessional care model for elderly patients in elective orthopaedic surgery: a study protocol for a prospective randomized controlled trial of a multimodal intervention in frail patients with hip and knee replacement

**DOI:** 10.1186/s12891-022-05955-w

**Published:** 2022-12-09

**Authors:** Tobias Kappenschneider, Günther Maderbacher, Markus Weber, Felix Greimel, Dominik Holzapfel, Lukas Parik, Timo Schwarz, Franziska Leiss, Michael Knebl, Jan Reinhard, Amadeus Dominik Schraag, Max Thieme, Agathe Turn, Julia Götz, Magdalena Zborilova, Loreto C. Pulido, Fady Azar, Jan-Frederik Spörrer, Britta Oblinger, Frederik Pfalzgraf, Leonie Sundmacher, Iryna Iashchenko, Sebastian Franke, Benedikt Trabold, Katrin Michalk, Joachim Grifka, Matthias Meyer

**Affiliations:** 1 Department of Orthopaedic Surgery, Regensburg University Medical Center, Bad Abbach, Germany; 2 Department of Orthopaedic and Trauma Surgery, Hospital Barmherzige Brüder Regensburg, Regensburg, Germany; 3 Department of Health Economics, Technical University of Munich, Munich, Germany; 4 Department of Anaesthesiology, Asklepios Klinikum Bad Abbach, Bad Abbach, Germany

**Keywords:** Orthogeriatric, Orthogeriatric co-management (OGC), Geriatric, Total hip arthroplasty (THA), Total knee arthroplasty (TKA), Elderly patients, Frailty, Multiprofessional care, Perioperative Care of Older Persons (POPS), Comprehensive geriatric assessment (CGA)

## Abstract

**Background:**

Due to demographic change, the number of older people in Germany and worldwide will continue to rise in the coming decades. As a result, the number of elderly and frail patients undergoing total hip and knee arthroplasty is projected to increase significantly in the coming years. In order to reduce risk of complications and improve postoperative outcome, it can be beneficial to optimally prepare geriatric patients before orthopaedic surgery and to provide perioperative care by a multiprofessional orthogeriatric team. The aim of this comprehensive interventional study is to assess wether multimorbid patients can benefit from the new care model of special orthopaedic geriatrics (SOG) in elective total hip and knee arthroplasty.

**Methods:**

The SOG study is a registered, monocentric, prospective, randomized controlled trial (RCT) funded by the German Federal Joint Committee (GBA). This parallel group RCT with a total of 310 patients is intended to investigate the specially developed multimodal care model for orthogeriatric patients with total hip and knee arthroplasty (intervention group), which already begins preoperatively, in comparison to the usual orthopaedic care without orthogeriatric co-management (control group). Patients ≥70 years of age with multimorbidity or generally patients ≥80 years of age due to increased vulnerability with indication for elective primary total hip and knee arthroplasty can be included in the study. Exclusion criteria are age < 70 years, previous bony surgery or tumor in the area of the joint to be treated, infection and increased need for care (care level ≥ 4). The primary outcome is mobility measured by the Short Physical Performance Battery (SPPB). Secondary outcomes are morbidity, mortality, postoperative complications, delirium, cognition, mood, frailty, (instrumental) activities of daily living, malnutrition, pain, polypharmacy, and patient reported outcome measures. Tertiary outcomes are length of hospital stay, readmission rate, reoperation rate, transfusion rate, and time to rehabilitation. The study data will be collected preoperative, postoperative day 1 to 7, 4 to 6 weeks and 3 months after surgery.

**Discussion:**

Studies have shown that orthogeriatric co-management models in the treatment of hip fractures lead to significantly reduced morbidity and mortality rates. However, there are hardly any data available on the elective orthopaedic care of geriatric patients, especially in total hip and knee arthroplasty. In contrast to the care of trauma patients, optimal preoperative intervention is usually possible.

**Trial registration:**

German Clinical Trials Register DRKS00024102. Registered on 19 January 2021.

**Supplementary Information:**

The online version contains supplementary material available at 10.1186/s12891-022-05955-w.

## Background

According to the United Nations (UN), the number of people aged 65 and older in Germany and worldwide will continue to rise in the coming decades [1]. In 2060, about 30% of the population in Germany will be over 65 years old and about 11% over 80 years old [2]. This demographic shift, along with improvements in general living standards, healthcare, nutrition and education, is leading to a steady increase in geriatric patients undergoing major surgeries [3, 4]. It is expected that the number of elderly patients with the need for elective orthopaedic surgery will continue to rise significantly. For example, the number of primary total hip and total knee arthroplasties (THA and TKA) is expected to increase by 71 and 85% respectively by 2030 [5].

However, the orthogeriatric patient is not a standard patient for whom the usual orthopaedic care is sufficient. Comorbidities, polypharmacy, frailty, osteosarcopenia, malnutrition, immobility, cognitive impairments and other geriatric syndromes as well as increased complication and mortality rates are typical for geriatric patients [6–8]. To address this challenge and improve care of elderly patients, centers have increasingly emerged in traumatology in recent years in which elderly patients are treated jointly by trauma surgeons and geriatricians. Orthogeriatric co-management (OGC) plays an important role especially in frail patients with hip and other fragility fractures today. Many studies have shown that orthogeriatric co-management models lead to significantly reduced morbidity and mortality rates in patients with hip fractures [9–14]. In addition, comprehensive geriatric care promoted functional improvement in older patients with hip fracture [15–17]. However, the models are country-specific and structurally different [18]. The transfer of these care models to other trauma-related conditions (spinal injuries or pelvic fractures) and their scientific investigation through studies is very limited so far [9, 19, 20].

In elective orthopaedic surgery, orthogeriatric co-management models are still less established. Only few studies have investigated the effect of OGC in the context of elective orthopaedic surgery [21]. However, the number of geriatric patients is increasing not only for hip fractures, but also for elective orthopaedic surgery, especially total joint replacement. Based on the favourable data in geriatric traumatology in the care of hip fractures, it is imperative to develop specific orthogeriatric co-management models for patients with total hip and knee arthroplasty and to investigate their benefits for patients. In contrast to the emergency treatment of hip fractures, a major advantage here can be the longer time for optimal preoperative preparation (prehabilitation). A randomized, blinded trial has shown that personalized prehabilitation in patients prior to elective major abdominal surgery significantly improves the physical performance of patients and also reduces postoperative complications by more than 50% [22]. Already at this stage, the geriatrician should examine the patient, a comprehensive geriatric assessment (CGA) should be done and appropriate preoperative interventions (API) should be arranged.

### Objectives and research hypothesis

The aim of our trial is to assess the effectiveness of comprehensive orthogeriatric care (SOG care model) versus standard orthopaedic care with treatment by orthopaedic surgeons only in patients undergoing primary THA and TKA. The SOG care model consists of a comprehensive geriatric assessment, appropriate preoperative intervention, fast-track surgery principle and multimodal perioperative care on a SOG unit. We examine the outcomes of randomly assigned patients with assessments on postoperative days 1 to 7, 4 to 6 weeks and 3 months after surgery. Because immobility is an immediate result of osteoarthritis (OA) and leads to long-term functional deterioration, we choose mobility as the primary outcome. The current report outlines the research design and protocol for evaluating this randomized controlled trial of a multimodal intervention in frail patients with hip and knee replacement.

We hypothesize that multimodal perioperative orthogeriatric co-management (SOG care model) can improve the mobility of patients with total hip and knee arthroplasty (measured by SPPB).

The objectives of the randomized controlled trial are.

1. The primary objective is to examine the impact of comprehensive geriatric assessment, appropriate preoperative intervention, fast-track surgery principle and multimodal perioperative care on a SOG unit (SOG care model) versus standard care after total hip and knee arthroplasty on mobility on postoperative day 3 and 7, 4 to 6 weeks and 3 months after surgery.
2. The secondary objectives are to explore potential efficacy of the comprehensive geriatric assessment, appropriate preoperative intervention, fast-track surgery principle and multimodal perioperative care on a SOG unit (SOG care model) versus usual orthopaedic care after total hip and knee arthroplasty on overall health, disability, morbidity, mortality, postoperative complications including delirium, cognition, mood, frailty, activities of daily living/instrumental activities of daily living, malnutrition, pain, polypharmacy, PROM and PREM on postoperative day 1 to 7, 4 to 6 weeks and 3 months after surgery.
3. The tertiary objectives are to test the effect of comprehensive geriatric assessment, appropriate preoperative intervention, fast-track surgery principle and multimodal perioperative care on a SOG unit (SOG care model) versus standard care after total hip and knee arthroplasty on length of hospital stay, readmission rate, reoperation rate, transfusion rate and time to rehabilitation.

In addition, within the framework of the study, we aim:

4. To develop and validate a new screening tool (SOG screening) for geriatric patients undergoing elective orthopaedic surgery.
5. To develop and validate a routine preoperative laboratory test for orthogeriatric patients.

## Methods/design

### Study design

**Fig. 1:**
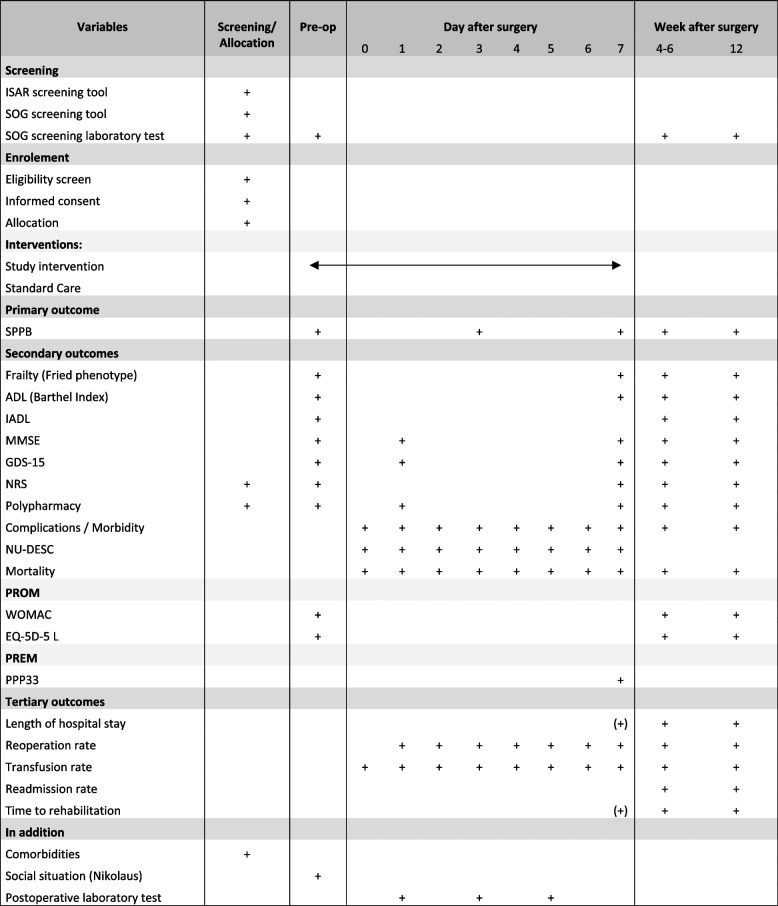
Standard Protocol Items Recommendations for Interventional Trials (SPIRIT) Schedule of enrolment, interventions, and assessments. SPPB: Short Physical Performance Battery, ADL: Activities of Daily Living, IADL: Instrumental Activities of Daily Living, MMSE: Mini-Mental State Examination, GDS: Geriatric Depression Scale, NRS: Nutritional Risk Screening, NU-DESC: Nursing Delirium Screening Scale, PROM: Patient Reported Outcome Measurement, WOMAC: Western Ontario and McMaster Universities Osteoarthritis Index, EQ-5D: Euroquol Quality of Life Index, PREM: Patient Reported Experience Measurement, PPP33: Perioperative Patient Questionnaire, ISAR: Identification of Seniors at Risk, SOG: Special Orthopaedic Geriatrics

The SOG study is a registered, monocentric, prospective, randomized controlled trial. This parallel group RCT with a total of 310 patients is intended to investigate the specially developed multimodal care model (SOG care model) for orthogeriatric patients with total hip and knee arthroplasty (intervention group), which already begins preoperatively, in comparison to the usual orthopaedic care without orthogeriatric co-management (control group). The study data will be collected preoperative, postoperative day 1 to 7, 4 to 6 weeks and 3 months after surgery. The duration of the study was originally planned for 3 years. Due to the Corona-19 pandemic, an extension to 3.5 years is expected to be necessary. The trial is registered at the German Clinical Trials Register and the registry platform for international clinical trials of the World Health Organization (WHO) under the same Main ID DRKS00024102. We used the Standard Protocol Items: Recommendations for Interventional Trials.

(SPIRIT) guidelines to guide the reporting of our trial protocol [23]. A SPIRIT Checklist is provided as Additional file 2, and a flow diagram is included as Fig. 1.

### Study setting

The study will be conducted at the Orthopaedic Department of Regensburg University Center, Asklepios Klinikum Bad Abbach, Germany. Here, about 18,000 patients are treated per year in the university outpatient clinic and > 1500 endoprosthetic procedures on the hip and knee joints are performed annually. Participants are recruited at the university outpatient clinic if they have a diagnosis of primary hip or knee osteoarthritis and an indication for THA or TKA. Preoperative interventions will be provided after randomization in written form or by telephone by the research nurse and/or geriatrician to the patient/family doctor/specialist. Pre-op exams and interventions can also be performed on-site at the university outpatient clinic. After surgery, the patients in the intervention group are treated in a special orthogeriatric ward (SOG unit) and the patients in the control group are treated in an ordinary orthopaedic ward of the orthopaedic university hospital.

### Eligibility criteria

Patients (or a representative) must provide written, informed consent before any study procedures occur. Included participants should fulfil the following inclusion criteria: primary hip or knee osteoarthritis, an age ≥ 70 years and multimorbidity or an age ≥ 80 years, with an indication for elective unilateral hip or knee replacement. The waiting time until surgery must be at least 4–6 weeks. Exclusion criteria: Age < 70 years, previous bony surgery or tumour in the area of the joint to be treated, acute infection and increased need for care (care level ≥ 4).

### Sample size

The sample size calculation is based on the primary endpoint of the analysis - “Improvement of Mobility”, measured by Short Physical Performance Battery (SPPB). According to previous studies, the clinically relevant difference between the two groups is conservatively estimated at 0,5 points with an expected standard deviation of 1,48 points [24]. Due to a 1:1 randomization, the number of cases per group is 139 to achieve a power of 80% at a 5% significance level (Gpower). Taking into account a possible dropout rate of 10%, a total of 310 patients should to be randomized.

The patient is not informed about the result of the randomization. The allocation is only known to the team directly treating the patient. In particular, the investigators who perform the post-intervention testing are blinded. For the patients, blinding is ensured by the fact that the patients in the control group also receive physiotherapy after surgery. Furthermore, the planned length of stay in hospital is the same for both study groups. An interdisciplinary ward round is simulated, but without orthogeriatric care in the actual sense. Very rare circumstances that may lead to emergency intervention during the study, and therefore possibly emergency unblinding, may include unpredictable highly pathological, life-threatening assessment, examination or laboratory results.

### Recruitment strategy

After the orthopaedic surgeons have assessed the patients referred for hip or knee problems in the university outpatient clinic and have given the indication for elective primary THA or TKA, the patient is presented to the research assistant and the geriatrician after checking the eligibility criteria. There will be a detailed explanation of the study with written informed consent. The research assistant assists the geriatrician in reviewing comorbidities and polypharmacy, and in conducting the Identification of Seniors at Risk (ISAR) screening [25], SOG screening and Nutritional Risk Screening (NRS) [26]. There is also a physical examination by the geriatrician and a laboratory test. Clinic administrators assign patients a surgery date in 4–6 weeks at the earliest or place patients on the surgery waiting list and assign them a surgery date later.

### Randomization and consent

If the eligibility criteria are met, the patient has signed the information and consent form and the date for the surgery has been set, the participants will be randomized into the intervention or control group (usual care group). The random allocation sequence is performed computer-aided using a professional online tool for randomized clinical trials called “Randomizer” (https://www.randomizer.at/, Randomizer Version 2.1.0, Institute for Medical Informatics, Statistics and Documentation, Medical University of Graz). To ensure a balanced allocation sequence, a stratified permuted block randomization with stratification by gender (female or male) and type of care (knee or hip) will be used. We do not stratify by age, as only geriatric patients are included into the trial (age ≥ 70). The block sizes will not be disclosed, to ensure concealment. Thus, randomization will be conducted without any influence of the principal investigators, physicians, therapists, statisticians, or the research assistant.

### The interventions

#### The SOG care model group (experimental group)

**Fig. 2:**
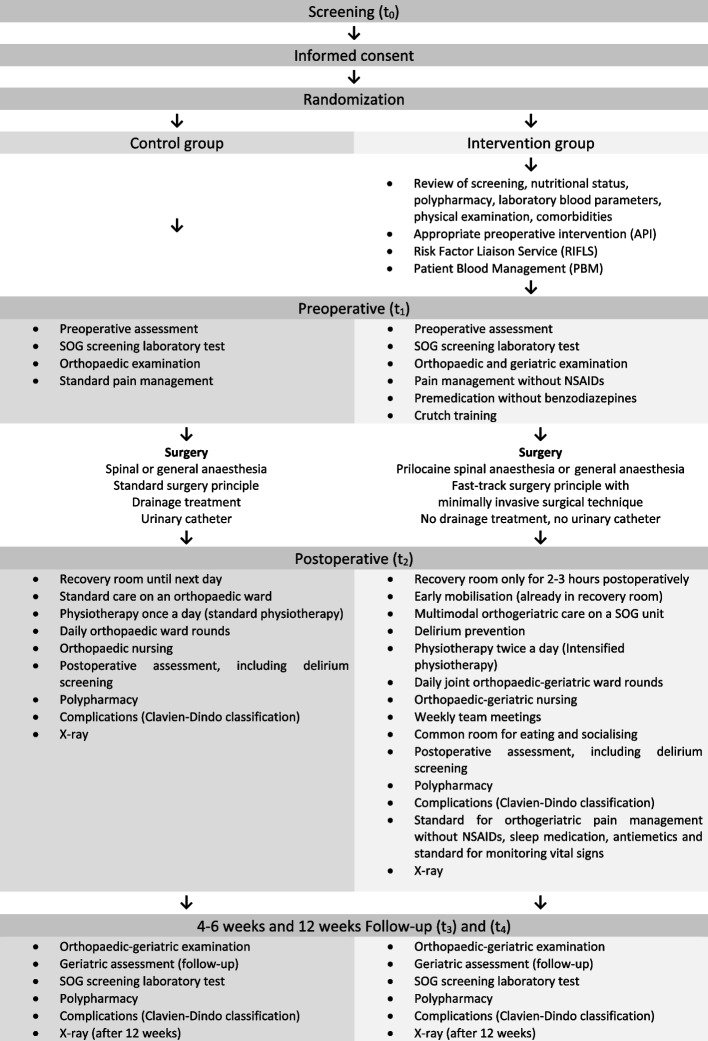
Study intervention and outcome assessments

The intervention and outcome assessments are summarized in Fig. 2.

### Screening

First, a screening is carried out to identify geriatric patients at increased risk of adverse health outcomes. For this purpose, the Identification of Seniors at Risk (ISAR) screening tool [25], which is used in hospital emergency departments, and a screening tool we developed specifically for elective orthopaedic surgery patients, the SOG screening tool, are used. Screening for malnutrition is performed by Nutritional Risk Screening (NRS) [26]. There is also a consultation with a geriatrician with physical examination and review of polypharmacy and comorbidities. The screening is completed by a SOG laboratory blood test.

### Appropriate preoperative intervention (API)

After reviewing the screening and assessment results, an appropriate preoperative intervention (API) is carried out. The API is aimed at the patient himself/herself, the general practitioner and, if applicable, the treating specialists. Contact with the participant and the external doctors can be made by telephone and/or in writing via doctor’s report/information material. Re-appointment to the university outpatient clinic is also possible. The API includes the Risk Factor Liaison Service (RiFLS) at the hospital. This addresses the modifiable risk factors of anaemia, malnutrition, vitamin D deficiency, obesity, diabetes mellitus and nicotine abuse. Depending on the comorbidities, physical examination findings and laboratory blood parameters, further diagnostic clarification is recommended or carried out. This also includes consultation with other specialists such as cardiologists, angiologists or neurologists. Optimization of polypharmacy with adjustment for age and comorbidities by the geriatrician, management of anticoagulation and patient blood management (PBM) is also part of the intervention before surgery.

### Preoperative/inpatient admission

A comprehensive geriatric assessment (CGA) consisting of Barthel Index [27], Instrumental Activities of Daily Living (IADL) scale [28], Social situation (Nikolaus) [29], Short Physical Performance Battery (SPPB) [30], Fried Frailty Phenotype [31], Mini Mental State Examination (MMSE) [32], Geriatric Depression Scale (GDS-15) [33] and NRS [26] is performed on admission. There is a physical examination by the orthopaedic surgeon and geriatrician and a SOG laboratory blood test. Perioperative pain management is adapted to older, multimorbid patients and does not include NSAIDs. Benzodiazepines are omitted from premedication. The patient already receives crutch training by physiotherapy before surgery.

### Perioperative/surgery

The patient is transported to the operating room and back to the ward by a nurse known to the patient. Aids such as glasses or hearing aids are given to the patient. Surgery is performed according to the fast-track surgery principle with prilocaine spinal anaesthesia or general anaesthesia and minimally invasive surgical techniques. Spinal anaesthesia is preferred. General anaesthesia is only used if the patient refuses spinal anaesthesia or it is not anaesthesiologically feasible. Redon drains and urinary catheters are not used.

### Postoperative management

After surgery, the patient remains in the recovery room for 2–3 hours for monitoring, unless complications arise or other reasons require longer monitoring. In the recovery room, early mobilisation is performed by physiotherapy and nursing. The patient is then transferred to a special orthogeriatric ward (SOG unit). The SOG unit is a specially equipped ward for elderly patients, where  special attention is paid to delirium prevention. There is also a common room for patients on the ward to eat and socialise. Postoperatively, the participant receives multimodal orthogeriatric care. This includes care by a multiprofessional orthogeriatric team on the ward, consisting of orthopaedic surgeon, geriatrician, activating-therapeutic nursing, intensified physiotherapy (twice a day), occupational therapist, psychologist, nutritional therapist and team-integrated medical social workers. There is a daily joint ward round by the orthopaedic surgeon, geriatrician and nursing. The entire orthogeriatric team meets once a week for a team meeting, where each individual patient is discussed on an interdisciplinary basis. A standardized screening for postoperative delirium is performed daily by the nursing staff using the Nursing Delirium Screening Scale (NU-DESC) [34]. Further postoperative geriatric assessment includes Barthel Index [27], SPPB [30], Fried Frailty Phenotype [31], Mini Mental State Examination (MMSE) [32], Geriatric Depression Scale (GDS-15) [33] and NRS [26]. A standardized blood laboratory test is performed on postoperative days 1, 3 and 5. Drug therapy is monitored and adjusted daily by the geriatrician. For the orthogeriatric ward, there are standards for pain management (without NSAIDs), sleep medication, antiemetics and standards for monitoring vital signs.

#### Control group

Patients in the control group receive standard care for total hip and knee arthroplasty. Pre-, peri- and postoperative care is provided according to the orthopaedic surgeon’s instructions. The standard surgical principle with the use of Redon drains and urinary catheters is performed. The patient remains in the recovery room until the following day. Mobilisation with physiotherapy takes place on the first day after surgery. Further inpatient care is provided on an orthopaedic ward. Postoperatively, physiotherapy is performed once a day. No other therapies are planned. The usual orthopaedic standards for pain management (with NSAIDs) and blood lab tests apply. Daily ward rounds are carried out by the orthopaedic surgeon and the orthopaedic nursing staff.

### Study outcomes

The study outcomes will be collected preoperative (baseline), postoperative day 1 to 7, 4 to 6 weeks and 3 months after surgery. Figure 1 summaries the primary, secondary and tertiary outcomes and measurement time.

### Primary outcome

*Short Physical Performance Battery (SPPB)* is a measure of physical functioning [30]. SPPB evaluates balance, mobility and muscle strength by examining an individual’s ability to stand in different positions, time to walk 4 m, and time to rise up from and sit down on a chair 5 times [30]. The tests are scored between 0 and 4, leaving a maximum score of 12 [30]. The SPPB is performed preoperatively on admission to hospital, on the 3rd day after surgery, on the 7th day after surgery before discharge from hospital, and at 4–6 weeks and 3 months after surgery.

### Secondary outcomes

Frailty will be assessed using *Fried Frailty Phenotype* which is composed of five items, three self-reported (unintentional weight loss, exhaustion and physical activity), and two performance-based items (strength (assessment based on the handgrip strength measurement) and speed). It is a widely used and validated frailty measure. Each item is scored 0 or 1 with a final score out of 5; higher scores indicate greater frailty [31, 35]. It is used preoperatively on admission to hospital, on the 7th day after surgery before discharge, at 4–6 weeks and 3 months after surgery.

The *Barthel Scale/Index* is an ordinal scale used to measure performance in activities of daily living (ADL). Ten variables describing ADL and mobility are scored, a higher number being a reflection of greater ability to function independently following hospital discharge [27]. It is used preoperatively on admission to the hospital, on the 7th day after surgery before discharge, at 4–6 weeks and 3 months after surgery.

The Lawton & Brody *Instrumental Activities of Daily Living Scale (IADL)* is an appropriate instrument to assess independent living skills [28]. These skills are considered more complex than the basic activities of daily living as measured by the Barthel Index. There are eight domains of function measured with the Lawton IADL scale. Participants are scored according to their highest level of functioning in that category. A summary score ranges from 0 (low function, dependent) to 8 (high function, independent) [28]. The IADL scale is performed on admission to hospital and at 4–6 weeks and 3 months after surgery.

The *Mini Mental State Examination (MMSE)* is a 30-point questionnaire that is used extensively in clinical and research settings to measure cognitive impairment. The test examines functions such as registration (repeating named prompts), attention and calculation, recall, language, ability to follow simple commands and orientation. Any score of 24 or more (out of 30) indicates a normal cognition. Below this, scores can indicate severe (≤9 points), moderate (10–18 points) or mild (19–23 points) cognitive impairment [32]. Testing is done preoperatively on admission to hospital, on the 1st postoperative day, on the 7th day after surgery before discharge, and after 4–6 weeks and after 3 months after surgery.

The 15-item *Geriatric Depression Scale (GDS-15)* is a short form of GDS and is used to screen, diagnose, and evaluate depression in elderly individuals. In scoring the GDS, 1 point is awarded for each answer that indicates depression. If a person scores more than 5 on the 15-question assessment, this may indicate the presence of depression [33]. The test is performed preoperatively on admission to hospital, on the 1st postoperative day, on the 7th day after surgery before discharge, and after 4–6 weeks and after 3 months after surgery.

The purpose of the *Nutritional Risk Screening (NRS)* system is to detect the presence of malnutrition and the risk of developing malnutrition in the hospital setting. It includes four questions as a pre-screening. If one of these is answered positively, a screening follows which includes surrogate measures of nutritional status, with static and dynamic parameters and data on the severity of the disease (stress metabolism). For each parameter, a score from 0 to 3 can result. Age over 70 years is considered as a risk factor, and is included in the screening tool as well, giving 1 point. A total score of ≥3 points means that the patient is at risk of malnutrition or already malnourished and therefore a nutritional therapy is indicated [26]. The NRS is assessed for the first time in the university outpatient clinic at the time of the screening. In addition, it is applied preoperatively on admission to hospital, on the 7th day after surgery and after 4–6 weeks and 3 months after surgery.

The *Nursing Delirium Screening Scale (Nu-DESC)* is an observational five-item scale for detecting delirium. The Nu-DESC has five dimensions with point values of 0 = non-existent, 1 = present and 2 = strongly present, the probability of delirium is given from a sum point value ≥ 2. It can thus be used as a metric scale of 0–10 with 10 = most severe delirium [34]. It is applied daily during inpatient stay.

The *Western Ontario and McMaster Universities Osteoarthritis Index (WOMAC)*, which is a pain index measurement for OA, is the most widely used parameter for knee joint function and also a tool for evaluating disorders related to OA of the lower extremities. The WOMAC consists of a total of 24 questions and three subscales. Among them, there are five questions related to pain, two questions related to stiffness, and 17 questions related to difficulties in performing activities of daily living in relation to physical function. The disease-specific tool is of use in clinical evaluation of changes in pain-related health status and clinical outcomes. The WOMAC is valid and reliable for defining function in lower extremity disorders [36, 37]. It is used preoperatively, 4–6 weeks and 3 months after surgery.

*Health and Quality of Life Questionnaire (EQ-5D-5 L)* measures the patient’s health-related quality of life. Five questions are scored on a 5-point scale. Additionally, the self-rated health is reported on a vertical, visual analogue scale [38, 39]. The measurements are taken preoperatively, 4–6 weeks and 3 months after surgery.

*PPP33* is a patient-oriented questionnaire with 33 items that allows participants to assess the quality of the perioperative period. Not only postoperative somatic disorders are addressed, but all relevant aspects of the entire perioperative period [40]. It is used on the 7th day after surgery before discharge from hospital.

Other secondary outcomes listed in Fig. 1 such as *polypharmacy*, *complications/morbidity (Clavien-Dindo classification)* [41] and *mortality* are collected in standardized forms. The time of data collection is presented in Fig. 1.

### Tertiary outcomes

The tertiary outcomes *length of hospital stay*, *reoperation rate*, *transfusion rate*, *rehospitalization rate* and *time to rehabilitation* are captured in the acute phase, during inpatient stay, and 4–6 weeks and 3 months after surgery.

### Sociodemographic - and other variables

Age, sex, adiposity (body mass index), smoking, medication list, anticoagulants, comorbidities, social situation (Nikolaus) [29], ISAR screening tool [25] and our SOG screening tool are variables captured at baseline. The SOG screening laboratory test includes C-reactive protein (CRP), complete blood count (CBC), Quick-%, international normalized ratio (INR), partial thromboplastin time (PTT), serum sodium, serum potassium, serum calcium, serum creatinine (Cr), blood urea nitrogen (BUN), creatinine clearance (Ccr), serum protein, serum albumin, total bilirubin, aspartate aminotransferase (AST), alanine aminotransferase (ALT), gamma glutamyltransferase (GGT), alkaline phosphatase, blood glucose level, glycosylated hemoglobin A (HbA1c), serum iron, ferritin, transferrin, transferrin saturation, thyroid-stimulating hormone (TSH), folate, vitamin B12, vitamin D, and at the time of hospital admission, an additional urinalysis.

### Adherence to the program

To ensure adherence to the program, the doctors, nurses and therapists involved in the project are trained and motivated. The research nurse accompanies the participants to all examinations such as laboratory tests and geriatric assessments that are required as part of the study. The doctors, nurses and therapists document the collected data directly in the electronic database GERD or in documentation sheets. This is checked by the research assistants in a protocol list. Already during the inpatient clinic stay, each participant receives the appointments for the follow-up examinations in writing.

### Adverse events

Adverse events or harm from any source will be reported to the research team and recorded on a structured form. Any serious or unexpected adverse events that occur during the study and may affect the safety of the study participants or the conduct of the study will be reported immediately in writing to the Ethics Committee.

### Data collection and management

Figure 1 provides an overview of the data collection timeline. Screening and follow-up examinations after 4–6 and 12 weeks take place in the university outpatient clinic. The preoperative assessment is carried out on the premises of the central patient admission of the orthopaedic university hospital.

All other peri−/postoperative data (day 0–7 after surgery) are collected on the wards of the orthopaedic university hospital. The study assessors received extensive training before the start of the study, in which they were individually trained on how to collect the study outcome measures in frail older people. All data will be stored at the research server at the hospital. Study data will be managed using electronic data capture tools. The study database will be password protected and kept on a secure network system. Passwords are changed at regular intervals. A complete back up of the database will be performed twice a week. A formal Data Monitoring Committee (DMC) is not required for this trial as risks are considered minimal.

### Trial management

The coordinating centre for the study is at the Department of Orthopaedic Surgery, Regensburg University Medical Center, Germany. The study coordinator and research assistants are responsible for submitting study documents, planning and conducting the study, collecting and managing data, receiving and storing consent forms, and publishing the study results. Statistical analysis will be performed by the Department of Health Economics, Technical University of Munich (TUM). The study is fully funded by the German Federal Joint Committee - GBA (Grant no. 01VSF19030 (SOG)) and the funding management is carried out by the German Aerospace Center. Research reports will be written every quarter and the appropriate use of the funding will be monitored in an annual interim report. The final results of this study are expected to be available in 2024.

### Data analysis

The evaluation is carried out as an intention-to-treat analysis. Baseline demographic and clinical characteristics of the participants will be described using summary statistics (means, standard deviations and frequencies). Significance in differences will be tested using t-test for continuous variables with normal distribution, chi-square test for categorical variables and Mann-Whitney U-test for continuous variables with non-normal distribution. Normality of the distributions will be tested graphically. We will examine the difference preoperatively and postoperatively (3 days, 7 days, 4–6 weeks, 3 months) with regard to the primary outcome variable (SPPB score) in the intervention and control group, as well as between the groups (postoperatively). To assess significance in differences we will use paired-samples t-test. Although the random assignment of the patients should provide unbiased results, the need for an adjusted analysis will be considered. To this end, in order to account for potential confounders, we will consider general linear models (e.g. multivariate linear regression and analysis of covariance [42] to evaluate between-group differences in SPPB scores. The need for an adjusted analysis within generalized linear (mixed) models will be considered as well. Secondary endpoints will be analysed both descriptively and standardized within adjusted regression analyses. *P*-values *p* < 0,05 will be considered as statistically significant. Detailed sensitivity analyses are planned for all outcomes of the intervention. Potentially missing data will be completed using adapted techniques (e.g. multiple imputation).

### Ethical considerations

The study was approved by the ethics committee of the University of Regensburg (2020/06/24, No. 20–1837-101). Participants will undergo an informed consent process and sign a consent form prior to randomization. Patients recruited for the project will also be offered usual care.

## Discussion

The number of older patients undergoing elective orthopaedic surgery is increasing dramatically due to demographic change, advances in surgery and anaesthesia, and shifts in patient expectations of healthcare. As a result of the growing ageing of the population and the associated increased prevalence for degenerative joint diseases, a significant increase in total hip and knee arthroplasties in particular is predicted [5, 43]. Orthogeriatric co-management in traumatology has prevailed in the care of elderly, multimorbid patients with hip fractures. These patients benefit from significantly reduced morbidity and mortality as well as improved functional outcome and mobility [10, 12, 13, 17, 44]. However, previous orthogeriatric co-management models from traumatology have focused primarily on interdisciplinary care after surgery and have limited transferability to the care of elective orthopaedic patients.

Therefore, national and international orthopaedic and trauma societies have been calling for years for an extension of orthogeriatric care models to other, non-trauma-related musculoskeletal diseases. In addition to multiprofessional postoperative care of geriatric patients in traumatology, several other aspects are decisive for elective orthopaedic surgery of elderly patients with non-trauma-related diseases. Optimal preoperative preparation and reduction of surgical risk of multimorbid patients are of great importance in elective orthogeriatric care.

First of all, a reliable screening tool is needed to identify orthogeriatric patients. The ISAR screening tool [25], which is used in hospital emergency departments, is not suitable for elective patients. It is too non-specific. For this purpose, a separate screening tool (SOG screening tool) has been developed and is currently evaluated in the SOG study. An elementary component of the SOG care model is the preoperative involvement of a geriatrician after identification of an orthogeriatric patient. In this way, the preoperative risk assessment, the comprehensive geriatric assessment (CGA) and an appropriate preoperative intervention (API) can take place at an early stage and already in an interdisciplinary manner before the planned surgery. These approaches have already shown a significant benefit in postoperative outcomes after major surgery as a sole intervention in studies [45–48]. The SOG care model addresses several aspects in addition to optimal preoperative preparation of the patient and complex multiprofessional care after surgery. Patient blood management, anaesthesia, perioperative drug therapy, anticoagulation, standardized laboratory tests, crutch training, catheter avoidance, SOG unit, delirium prevention/delirium screening, orthopaedic-geriatric nursing, orthopaedic-geriatric ward rounds and common room for eating and socialising are further important components of the SOG care model. Another decisive element of the SOG concept is the surgical technique. Total hip and knee arthroplasty is carried out according to fast-track surgery principles, resulting in less pain and enabling early mobilisation. All these individual components together form the SOG care model.

We hypothesize that multimodal perioperative orthogeriatric co-management (SOG care model) can improve the mobility of patients with total hip and knee arthroplasty (measured by SPPB). In addition, further improvements in postoperative complications, delirium, cognition, mood, frailty, activities of daily living/instrumental activities of daily living, malnutrition, polypharmacy, length of hospital stay, rate of readmission to hospital, blood transfusion rate and other variables can be expected as a result of the multimodal intervention.

The proposed study has some limitations. Participant recruitment will take place within one hospital site, which may limit its generalizability to other hospital care settings. SOG investigators have considered the challenge of applicability to other settings during the study protocol development. The SOG care model is a complex, interdisciplinary intervention, but it also requires a functioning interaction of different professional groups in the hospital. A further limitation is the structurally impossible blinding of the staff on whose wards the concept is implemented. However, this could rather lead to a more preventive treatment of patients in the control group and to better results in standard orthopaedic care.

A major strength is the prospective randomized study design with 310 participants. The single centre model has the advantage that all patients are treated in the same way, thus reducing possible confounders. Other strengths of our proposed study include the use of valid and reliable measurements and the interdisciplinary involvement of all key professional groups involved in the implementation process. Some outcome measures can be compared or confirmed with other measures collected in the study. The primary outcome is physical functioning as measured by SPPB [30]. The SPPB has also been used in prior research to measure effects of interventions on physical functioning. In one study the SPPB was used to examine the effect of comprehensive geriatric care, compared to orthopaedic care, in the acute postoperative phase during hospital stay [44]. The SPPB was responsive and able to detect a difference between both groups even a few days after surgery. Our primary outcome measure is therefore considered to be sensitive to change, responsive and also used in other comparable studies. Our results are expected to be comparable to others, and this will strengthen the validity of the study. Measures with already known evidence-based benefits in the treatment of orthogeriatric patients with hip fractures were considered. PROM and PREM are also part of the study. The data analysis is carried out externally and independently at the Department of Health Economics, Technical University of Munich.

Medical Research Council (MRC) criteria define a “complex intervention” as interventions that are built up from a number of components, which may act both independently and inter-dependently [49]. These components include behaviors, behavior parameters, and methods of organizing those behaviors, and they may have an effect at the individual patient level, organizational, or service level or population level (or all of these in some cases) [49]. The present experimental intervention is considered to be a complex intervention. The outline for the arguments in this protocol is organized according to the guidelines of the Medical Research Council (MRC) guidance on how to develop and evaluate complex interventions [49].

The results of this prospective randomized controlled trial may, if successful, lead to the costs of the SOG care model being covered by health insurers in Germany and hospitals being able to use the SOG care model for orthogeriatric patients with knee and hip replacements, but also for other non-trauma-related musculoskeletal diseases. The results of this study will be presented as soon as they become available.

### Trial status and time plan of the study

The protocol has the version number 1, dated 26th October 2021, the recruitment began in April 2021 and will probably be completed by October 2023 (The study is being extended due to the COVID-19 pandemic). Participant follow-up and data collection will continue for 3 months after recruitment. For the subsequent data analysis, 5 months are calculated. Thereafter, we will write up and publish peer-reviewed articles.

## Supplementary Information

**Additional file 1.** SOG Screening Tool.

**Additional file 2.** SPIRIT Checklist.

**Additional file 3.** Administrative Information.

## Data Availability

Dissemination strategies include reports, the presentation of results in peer-reviewed journals and at conferences, and public relations activities in line with the recommendations of the International Committee of Medical Journal Editors (ICMJE). Due to data protection restrictions and regulations only the University of Regensburg and the Technical University of Munich will have access to the full dataset. The datasets generated and/or analysed during the current study are available on reasonable request due to privacy or other restrictions.

## Abbreviations

ADL: Activities of daily living
ALT: Alanine aminotransferase
API: Appropriate preoperative intervention
AST: Aspartate aminotransferase
BUN: Blood urea nitrogen
CBC: Complete blood count
CGA: Comprehensive geriatric assessment
Cr: Creatinine
Ccr: Creatinine clearance
CRP: C-reactive protein
EQ-5D: Euroquol Quality of Life Index
GDS: Geriatric Depression Scale
GGT: Gamma glutamyltransferase
HbA1c: Glycosylated hemoglobin A
IADL: Instrumental activities of daily living
INR: International normalized ratio
ISAR: Identification of Seniors at Risk
MMSE: Mini Mental State Examination
MRC: Medical Research Council
NRS: Nutritional Risk Screening
NU-DESC: Nursing Delirium Screening Scale
OA: Osteoarthritis
OGC: Orthogeriatric co-management
PBM: Patient Blood Management
POPS: Perioperative Care of Older Persons
PREM: Patient-reported experience measures
PROM: Patient-reported outcome measures
PTT: Partial thromboplastin time
RCT: Randomized controlled trial
RiFLS: Risk Factor Liaison Service
SPPB: Short Physical Performance Battery
SOG: Special orthopaedic geriatrics
THA: Total hip arthroplasty
TKA: Total knee arthroplasty
TSH: Thyroid-stimulating hormone
TUM: Technical University of Munich
UN: United Nations
WOMAC: Western Ontario and McMaster Universities Osteoarthritis Index
