# Supplementary material for: Special orthopaedic geriatrics (SOG) - a new multiprofessional care model for elderly patients in elective orthopaedic surgery: a study protocol for a prospective randomized controlled trial of a multimodal intervention in frail patients with hip and knee replacement
Source: BMC Musculoskelet Disord. 2022 Dec 9;23:1079. doi: 10.1186/s12891-022-05955-w (PMC9733347; doi:10.1186/s12891-022-05955-w)
Supplement: Supplementary file 1 — Additional file 1. SOG Screening Tool. [file 12891_2022_5955_MOESM1_ESM.docx]

**SOG Screening Tool**

| **Please answer yes or no to each of these questions** | **Yes**  **1 Point** | **No**  **0 Point** |
| --- | --- | --- |
| 1. Do you suffer from loss of appetite? |  |  |
| 2. Do you regularly eat less than 3 meals a day? |  |  |
| 3. Have you lost weight recently? |  |  |
| 4. Do you regularly feel exhausted or powerless? |  |  |
| 5. Do you suffer from dizziness? |  |  |
| 6. Do you feel unsteady when walking or do you need aids? |  |  |
| 7. Have you had a fall in the past year? |  |  |
| 8. Do you have chronic wounds? |  |  |
| 9. Do you have pain at rest? |  |  |
| 10. Are you in need of care or do you need help in everyday life? |  |  |
| 11. Do you have problems with memory, with recall? |  |  |
| 12. Are you often sad or anxious? |  |  |
| 13. Do you sleep badly? |  |  |
| 14. Have you been hospitalized within the last 6 months? |  |  |
| 15. Do you take 5 or more different medicines a day? |  |  |
| **Total answers with yes** |  |  |
| **Evaluation of the geriatric screening:**  “**Yes**” answer is given 1 point, and “**No**” answer is given 0 point  **≥ 4 points**: geriatric screening positive | 🞏 Yes | 🞏 No |
