# Supplementary material for: Special orthopaedic geriatrics (SOG) - a new multiprofessional care model for elderly patients in elective orthopaedic surgery: a study protocol for a prospective randomized controlled trial of a multimodal intervention in frail patients with hip and knee replacement
Source: BMC Musculoskelet Disord. 2022 Dec 9;23:1079. doi: 10.1186/s12891-022-05955-w (PMC9733347; doi:10.1186/s12891-022-05955-w)
Supplement: Supplementary file 3 — Additional file 3. Administrative Information. [file 12891_2022_5955_MOESM3_ESM.docx]

**Administrative Information**

| **Title** | **Special orthopaedic geriatrics (SOG) - a new multiprofessional care model for elderly patients in elective orthopaedic surgery: a study protocol for a prospective randomized controlled trial of a multimodal intervention in frail patients with hip and knee replacement** |
| --- | --- |
| **Short Title** | SOG Trial |
| **Trial registration** | German Clinical Trials Register DRKS00024102 (also WHO trial registration with the same registration number) |
| **Date of registration** | 19/01/2021 |
| **Protocol version** | Issue Date: 26th October 2021, Version 1 |
| **Study type** | Prospective, randomized, parallel-group, controlled trial |
| **Study duration** | 3.5 years (The study is being extended due to the COVID-19 pandemic) |
| **Single or multicenter design** | Single-center |
| **Funding** | The study is fully funded by the German Federal Joint Committee - GBA (Grant no. 01VSF19030 (SOG))  Contact name: Verena Dykstra  Address: Heinrich-Konen-Straße 1, 53227 Bonn (Germany)  Email: [verena.dykstra@dlr.de](mailto:verena.dykstra@dlr.de) |
| **Role of sponsor** | The funding source has no influence over the study design, implementation, evaluation or dissemination of the findings of the study. The authors and their contributions to the manuscript are independent from the funder. |
| **Ethics** | The protocol was reviewed and approved by the ethics committee of the University of Regensburg (2020/06/24), No. 20-1837-101 |
| **Author details** | ^1^Department of Orthopaedic Surgery, Regensburg University Medical Center, Germany  ^2^Department of Orthopaedic and Trauma Surgery, Hospital Barmherzige Brüder Regensburg, Germany  ^3^Department of Health Economics, Technical University of Munich, Germany  ^4^Department of Anaesthesiology, Asklepios Klinikum Bad Abbach, Germany |
| **Contact for scientific queries** | Joachim Grifka, Department of Orthopaedic Surgery, Regensburg University Medical Center  Tobias Kappenschneider, Department of Orthopaedic Surgery, Regensburg University Medical Center, email: t.kappenschneider@asklepios.com |
| **Authors Contributorship** | *TK, MM, GM*, *LS* and *JG* conceived of the study. *FG, DH, TS* and *BT* initiated the study design and AT, *FL, MK, MZ, JR, ADS, LP, MT, JG, LCP, FA, JFS, BO* and *KM* helped with implementation. *LS, SF* and *II* provided statistical expertise in clinical trial design and they are conducting the primary statistical analysis. All authors contributed to refinement of the study protocol and approved the final manuscript |
| **Public title** | Special orthopaedic geriatrics (SOG) - a new multiprofessional care model for elderly patients in elective orthopaedic surgery |
| **Scientific title** | Special orthopaedic geriatrics (SOG) - a new multiprofessional care model for elderly patients in elective orthopaedic surgery: a study protocol for a prospective randomized controlled trial of a multimodal intervention in frail patients with hip and knee replacement |
| **Country of recruitment** | Germany |
| **Health conditions and problems studied** | Orthogeriatric co‑management, elderly patients in elective orthopaedic surgery, perioperative care of older persons, multiprofessional care, hip and knee replacement, postoperative complications |
| **Intervention** | Primary total hip and knee arthroplasty with comprehensive geriatric assessment (CGA), appropriate preoperative intervention (API), fast-track surgery principle and multimodal orthogeriatric care on a SOG unit (SOG care model)  Primary total hip and knee arthroplasty with standard care |
| **Key inclusion and exclusion criteria** | **Inclusion criteria:** Primary hip or knee osteoarthritis, age ≥ 70 years and multimorbidity or age ≥ 80 years with indication for hip and knee replacement  **Exclusion criteria:**Age < 70 years, previous bony surgery or tumour in the area of the joint to be treated, acute infection, increased need for care (care level ≥ 4) |
| **Objectives** | **1.** Improving the quality of care and addressing deficits in care for elderly orthopaedic patients with multimorbidity  **2**. Measuring the impact of comprehensive geriatric assessment, appropriate preoperative intervention, fast-track surgery principle and multimodal perioperative care on a SOG unit (SOG care model) versus standard care on overall health and disability, morbidity and mortality, postoperative complications including delirium, cognition and mood, mobility and frailty, activities of daily living/instrumental activities of daily living, malnutrition and pain, polypharmacy, PROM and PREM postoperative day 1 to 7, 4 to 6 weeks and 3 months postoperative  **3.** Testing the effect of comprehensive geriatric assessment, appropriate preoperative intervention, fast-track surgery principle and multimodal perioperative care on a SOG unit (SOG care model) versus standard care on length of hospital stay, readmission rate, reoperation rate, transfusion rate and time to rehabilitation  **4.** Development and validation of a new screening tool (SOG screening) for geriatric patients in elective orthopaedic surgery  **5.** Development and validation of a routine preoperative screening laboratory test |
| **Primary outcome measure; hypothesis** | Mobility; multimodal perioperative orthogeriatric co-management can improve the mobility of patients with total hip and knee arthroplasty (measured by SPPB) |
| **Secondary outcome measures** | Morbidity, mortality, postoperative complications, delirium, cognition, mood, frailty, activities of daily living (ADL)/instrumental activities of daily living (IADL), malnutrition, pain, polypharmacy, PROM, PREM |
| **Tertiary outcome measures** | Length of stay, readmission rate, reoperation rate, transfusion rate, time to rehabilitation |
| **Target sample size** | 310 |
| **Recruitment status** | Recruiting |
| **Statistical analysis** | Data analysis is performed by the Department of Health Economics, Technical University of Munich. The evaluation is carried out as an intention-to-treat analysis. The exact type of statistical analysis is described in detail in Methods/Design under Data analysis. |
